# OpenNotes in dentistry as a strategy for patient engagement: opportunities, barriers, and a framework for implementation

**DOI:** 10.3389/froh.2026.1846849

**Published:** 2026-09-22

**Authors:** Toshiyuki Nagasawa, Osamu Uehara, Hirofumi Matsuoka, Katsuya Kawanishi, Izabela Mamcarz, Renata Chałas, Piotr Stachurski, Kamil Torres, Tsuneo Sekimoto, Yoshihiro Abiko

**Affiliations:** 1 Division of Periodontology and Endodontology, Department of Oral Rehabilitation, School of Dentistry, Health Sciences University of Hokkaido, Tobetsu, Hokkaido, Japan; 2 Division of Advanced Clinical Education, Department of Integrated Dental Education, School of Dentistry, Health Sciences University of Hokkaido, Tobetsu, Hokkaido, Japan; 3 Division of Disease Control and Molecular Epidemiology, Department of Oral Growth and Development, School of Dentistry, Health Sciences University of Hokkaido, Tobetsu, Hokkaido, Japan; 4 Simulation Laboratory for Patient Safety, Chair of Medical Education, Medical University of Lublin, Lublin, Poland; 5 Department of Oral Medicine, Medical University of Lublin, Lublin, Poland; 6 Department of Dental Emergency, Medical University of Lublin, Lublin, Poland; 7 Department of Plastic, Reconstructive Surgery and Microsurgery, Medical University of Lublin, Lublin, Poland; 8 The Nippon Dental University School of Life Dentistry at Niigata, Niigata, Japan; 9 Division of Oral Medicine and Pathology, Department of Human Biology and Pathophysiology, School of Dentistry, Health Sciences University of Hokkaido, Tobetsu, Hokkaido, Japan

**Keywords:** dental informatics, dental records, electronic health records, online record access, OpenNotes, patient engagement, shared decision-making, teledentistry

## Abstract

Patient engagement is increasingly recognized as a cornerstone of high-quality, patient-centered care. Although the OpenNotes initiative, which gives patients direct access to their clinical notes through electronic health records, has demonstrated significant benefits in medicine, its application in dentistry remains largely unexplored despite the elective and decision-intensive nature of dental care. This targeted narrative review was informed by a PubMed search conducted on March 26, 2026, using the terms (“OpenNotes” OR “shared clinical notes” OR “patient access to records”) AND (“dentistry” OR “dental”), which identified 12 articles and no studies directly addressing OpenNotes implementation in dental practice. Consequently, the anticipated benefits for dentistry are derived by analogy with medical evidence and should be regarded as hypotheses requiring empirical testing rather than established findings. We examined the potential of OpenNotes as a patient engagement strategy in dentistry, including its benefits, the structural and technical barriers unique to dental documentation, and the enabling role of emerging technologies such as artificial intelligence and teledentistry, and found that, based on evidence from medical OpenNotes programs, note sharing may improve treatment adherence, shared decision-making, and patient empowerment in dentistry as well, although important barriers remain, including procedure-centric documentation, specialized terminology, fragmented electronic health record adoption, and privacy regulations; overall, OpenNotes appears to hold substantial promise for enhancing patient engagement in dentistry, but realizing this potential will require coordinated efforts in documentation standardization, clinician training, technology infrastructure, and regulatory compliance, with pilot implementation in academic dental institutions representing a pragmatic first step toward building an evidence base for broader adoption.

## 1 Introduction

Patient engagement—encompassing informed participation, shared decision-making, and active self-management—has emerged as a critical dimension of contemporary healthcare quality. Engaged patients are more likely to adhere to treatment plans, report higher satisfaction, and achieve better health outcomes (1). In dentistry, where the majority of interventions are elective and frequently involve multiple treatment alternatives, patient engagement is not merely desirable but arguably indispensable to ethical and effective care.

The OpenNotes initiative, launched in the United States in the early 2010s, operationalizes patient engagement through a simple but transformative mechanism: granting patients unrestricted access to their clinicians' visit notes via electronic health records (EHRs). Large-scale studies in medicine have demonstrated that OpenNotes increases treatment adherence, facilitates error detection, and strengthens the therapeutic alliance between patients and providers (2, 3). The incorporation of OpenNotes principles into U.S. federal policy through the 21st Century Cures Act—enacted in 2016 and implemented through the 2020 ONC final rule on interoperability and information blocking—formalized this paradigm shift toward documentation transparency (4).

Despite these developments, the application of OpenNotes principles to dental practice remains largely unexplored. Dentistry presents a distinct documentation landscape, characterized by procedure-centric records, specialized terminology, and heterogeneous EHR adoption, all of which may impede straightforward implementation. At the same time, the elective and decision-intensive nature of dental care—where patients routinely face choices among restorative, prosthetic, and surgical alternatives—positions dentistry as a domain particularly amenable to engagement-enhancing transparency.

This paper examines OpenNotes as a patient engagement strategy within the dental context. We review the evidence base from medical OpenNotes programs, analyze structural and technical barriers specific to dental documentation, explore the enabling potential of emerging technologies, and propose a practical framework for pilot implementation. Our aim is to advance a theoretically grounded and operationally feasible agenda for OpenNotes-driven patient engagement in dentistry.

This narrative review was informed by a targeted literature search conducted in PubMed on March 26, 2026. The search strategy combined the following terms using Boolean operators: (“OpenNotes” OR “shared clinical notes” OR “patient access to records”) AND (“dentistry” OR “dental”). No date restrictions were applied. The search retrieved 12 articles. Titles and abstracts were screened by two authors independently for relevance to patient engagement, dental documentation, EHR systems, and digital health infrastructure in dentistry. None of the articles directly examined OpenNotes implementation in dental practice. Because PubMed's automatic term mapping and phrase indexing may alter the interpretation of untagged queries over time, the search was re-executed on July 4, 2026, restricted to title and abstract fields and using proximity syntax for phrases absent from PubMed's phrase index: [“OpenNotes”[tiab] OR “shared clinical notes”[tiab:∼0] OR “patient access to records”[tiab]] AND [dentistry[tiab] OR dental[tiab]]. This field-restricted search retrieved no articles, consistent with the absence of published studies directly examining OpenNotes in dental settings; the larger yield of the original untagged search reflects PubMed's automatic term mapping across all indexed fields. Given the absence of a dental-specific evidence base, we additionally searched for evidence from medical OpenNotes programs (search terms: “OpenNotes” OR “patient portal” OR “shared notes” AND “patient engagement” OR “adherence” OR “shared decision-making”), prioritizing large-scale prospective studies and policy documents. The narrative synthesis presented herein is necessarily exploratory and is subject to the limitations of non-systematic evidence selection.

## 2 Patient engagement and OpenNotes: evidence from medicine

Patient engagement is a multidimensional construct encompassing cognitive, emotional, and behavioral components of patient participation in healthcare. Engaged patients demonstrate greater understanding of their diagnoses and treatment options, higher adherence to prescribed regimens, more effective self-management, and more productive interactions with clinicians (1). OpenNotes operationalizes engagement through the provision of direct, unrestricted access to clinical visit notes, transforming documentation from a clinician-facing administrative tool into a shared communicative resource.

The foundational study by Delbanco et al. (2), a quasi-experimental investigation across three U.S. health systems, demonstrated that a large majority of patients who accessed their notes reported improved understanding of their health conditions, greater recall of clinical information, and an enhanced sense of control over their care. Importantly, the majority of participating clinicians reported no significant increase in workload, and concerns regarding patient distress or litigation were largely not substantiated.

Subsequent longitudinal data have reinforced and extended these findings. Walker et al. (3), reporting seven-year follow-up data from OpenNotes participants, documented sustained improvements in patient-reported engagement, with patients describing their notes as valuable tools for managing medications and preparing for clinical encounters. Bell et al. (5) demonstrated that note access facilitated the detection of documentation errors and safety concerns by patients themselves, introducing a novel mechanism for patient participation in clinical safety. Notably, benefits were amplified among patients with chronic conditions, those with limited English proficiency, and family caregivers managing care on behalf of vulnerable individuals (3).

From a policy perspective, the 21st Century Cures Act (enacted in 2016), as implemented through the ONC final rule published in 2020, mandated that U.S. healthcare providers offer patients free and timely electronic access to a broad range of clinical notes, including progress notes, discharge summaries, and consultation reports. The Office of the National Coordinator for Health Information Technology (ONC) enforced these provisions to promote information transparency and reduce information blocking (4). This legislative milestone reflects a broader societal consensus that patient access to clinical documentation is a fundamental component of patient-centered care.

It is important to note that the term “OpenNotes” itself reflects a culturally specific framing originating in the United States. At the international level, the broader, solution-neutral concept of Online Record Access (ORA)—encompassing any mechanism by which patients may access their clinical records electronically—has been proposed as a more inclusive descriptor. This distinction has been made explicit by the NORDeHEALTH research consortium, which has examined patient online record access across Nordic and Baltic healthcare systems and documented substantial variation in how such access is conceptualized, implemented, and experienced across national contexts (16). Sweden provides a particularly instructive example: the national patient portal 1177.se offers patients comprehensive access to their clinical records, including visit notes, through the Journalen system—effectively implementing the substance of OpenNotes without adopting that terminology. This observation suggests that the implementation evidence base for patient record access is considerably broader than the OpenNotes literature alone, and that terminology and national context should be considered when evaluating the applicability of findings across healthcare systems.

Qualitative and quantitative studies from the Swedish context offer complementary perspectives to the U.S.-derived OpenNotes evidence base. Research examining patients' attitudes toward record transparency in Sweden has documented patients' concerns about the sensitivity of disclosed information that parallel those documented in North American settings (16). Separately, cross-sectional studies of public trust in the handling of electronic health data in Sweden suggest that population-level acceptance of digital record access may be shaped by pre-existing institutional trust in healthcare systems—a contextual factor that may differ substantially between Nordic and other national settings, including Japan (17). These findings underscore the importance of contextualizing implementation evidence within the specific institutional, cultural, and regulatory environment in which dental OpenNotes programs are to be developed.

Collectively, the medical evidence supports OpenNotes as a robust patient engagement strategy, with demonstrable benefits across diverse patient populations and clinical contexts. The translation of this strategy to dentistry, however, requires careful consideration of the structural and contextual differences that distinguish dental documentation from its medical counterpart. The established medical evidence in each major outcome domain, the corresponding proposed dental applications, and the evidence status of each anticipated outcome are summarized in Table 1.

**Table 1:** Comparison of established medical OpenNotes outcomes with proposed dental applications and expected outcomes. Evidence status is indicated for each domain: findings based on medical evidence are extrapolated to dentistry unless otherwise noted. Red-shaded rows indicate domains where evidence from medicine identifies potential risks requiring monitoring.

Domain	Medical Evidence (established)	Proposed Dental Application	Expected Outcome (evidence status)
Treatment adherence & self-management	Improved medication adherence (60%–78%); better chronic disease self-management	Periodontal maintenance; orthodontic appliance compliance; preventive home-care	Improved compliance anticipated (extrapolated); unvalidated in dental settings
	Delbanco et al. (2); Walker et al. (3)		
Comprehension & recall of clinical information	Improved recall of diagnoses and instructions; enhanced health understanding	Multi-stage dental treatment plan review; recall of alternatives discussed during consultation	Enhanced comprehension anticipated; particularly relevant for complex restorative/surgical planning (not yet studied)
	Delbanco et al. (2); DesRoches et al. (8)		
Shared decision-making	Greater sense of control; preparation for clinical encounters; increased engagement	Documentation of options discussed (implant vs. bridge vs. denture; endo vs. extraction); patients revisit reasoning after consultation	Enhanced autonomous decision-making anticipated; alignment with informed consent ethics in elective care (extrapolated)
	Delbanco et al. (2); Walker et al. (3)		
Error detection & patient safety	Patients identified documentation errors and safety concerns in their own records	Detection of incorrect tooth notation, medication allergies, or treatment sequencing errors in dental records	Plausible; dental records contain structured data susceptible to transcription error; no dental-specific evidence
	Bell et al. (5)		
Caregiver & proxy support	Family caregivers reported enhanced ability to support patients; greatest benefit in limited-English-proficiency and chronic illness groups	Caregivers of paediatric, elderly, or cognitively impaired dental patients; support for care coordination across providers	Anticipated benefit, particularly in paediatric dentistry and special needs care (extrapolated; context-dependent)
	Walker et al. (3)		
Continuity of care across providers	Patients could share notes with other clinicians; reduced information loss at care transitions	Multi-provider dental episodes (referral to specialist; transitions between public and private care)	Anticipated improvement in information continuity; relevant in referral-heavy care pathways (not yet studied)
	Walker et al. (3)		
Clinician workload	Minimal increase reported; 0%–5% longer consultations; 0%–8% increased after-hours queries; concerns largely not substantiated post-implementation	Unknown in dental settings; potential concerns include increased patient queries about radiographic findings and complex notation	Workload impact uncertain; AI-assisted summarisation may mitigate additional burden; pilot data required
	Delbanco et al. (2)		
Documentation candour	Some evidence of avoidance of speculative entries; mixed findings across settings	Dental notes document uncertain findings (incipient caries under observation; provisional diagnoses); risk of note modification under patient scrutiny	Potential risk; requires monitoring in dental pilots; may be context-specific
	Blease et al. (18); Turner et al. (19)		

## 3 Dental documentation as a barrier to patient engagement

The structure and culture of dental documentation present distinct challenges to the implementation of OpenNotes as a patient engagement strategy. Understanding these barriers is a prerequisite for designing effective implementation approaches.

### 3.1 Procedure-centric and highly technical documentation

Dental records are primarily organized around procedures and anatomical structures rather than patient narratives or clinical reasoning processes. Notes routinely employ specialized abbreviations, procedural codes, and technical indices—such as the DMFT index, probing depth notation, and occlusal classification systems—that carry little meaning to non-specialist readers (6). Unlike medical progress notes, which are often structured around patient-reported symptoms and clinical reasoning, dental notes may consist primarily of procedural entries without contextual explanation. This documentation style, while efficient for clinical communication, represents a significant barrier to patient comprehension and thus to the engagement-enhancing potential of note access.

### 3.2 Image-heavy and interpretation-dependent records

Dental records incorporate substantial quantities of visual data, including periapical and panoramic radiographs, cone-beam computed tomography images, intraoral photographs, and periodontal charts. While these data are diagnostically central, they require professional interpretation to be clinically meaningful. Uncontextualized access to such material may engender misinterpretation, unnecessary anxiety, or unwarranted concern among patients encountering findings without explanatory guidance. This challenge has no direct parallel in standard medical note-sharing programs.

### 3.3 Fragmented and non-standardized EHR adoption

In contrast to the relatively advanced integration of EHR systems in medicine, dental practice exhibits wide variability in digital record-keeping (7). A significant proportion of dental practices continue to use paper-based systems or proprietary digital platforms that lack interoperability with broader health information networks. This fragmentation complicates any initiative requiring standardized, system-wide patient-facing note access. The absence of a universal dental data standard comparable to HL7 FHIR in medicine further impedes technical implementation.

### 3.4 Verbal communication as the primary mode of patient information

Clinical communication in dentistry is predominantly delivered verbally, chairside, at the point of care. Comprehensive written summaries for patients following dental encounters—analogous to after-visit summaries in medicine—remain uncommon in many settings. This communication pattern, while allowing for immediate interaction, limits patients' ability to review, reflect upon, or share clinical information after the encounter, reducing opportunities for engagement between visits. OpenNotes offers a structural remedy to this limitation by providing a durable, accessible record of clinical encounters.

## 4 OpenNotes as a patient engagement strategy in dentistry

The following subsections describe potential benefits of OpenNotes for dental patients. In the absence of dental-specific evidence — no published studies examining OpenNotes implementation in dental practice were identified in our literature search — each anticipated benefit is derived by analogy from the medical OpenNotes literature. Patient populations, documentation cultures, and care delivery models differ substantially between medicine and dentistry, and the magnitude, or indeed the direction, of engagement effects in dental OpenNotes programs cannot be assumed to replicate those observed in medical settings. Empirical validation through pilot implementation research is therefore a prerequisite for evidence-based adoption.

### 4.1 Enhancing comprehension and recall

Dental treatment plans frequently involve complex, multi-stage interventions with multiple decision points—such as choices among endodontic treatment, extraction, or restorative alternatives—that patients are expected to evaluate and consent to under time pressure. Evidence from medicine demonstrates that note access improves recall of clinical information and supports informed consent processes (2). By analogy with these medical findings, access to post-visit clinical notes in dentistry may meaningfully enhance comprehension and recall of these discussions—enabling patients to review treatment rationale and alternatives at their own pace—and support a more deliberate, unhurried informed consent process — though direct evidence from dental settings is not yet available.

### 4.2 Supporting shared decision-making

Dental care is quintessentially elective and decision-intensive. For a single missing tooth, patients may face choices among dentures, fixed bridges, and implant-supported restorations, each associated with differing clinical, financial, and temporal burdens. OpenNotes could support shared decision-making by providing patients with written documentation of the options discussed, their respective advantages and limitations, and the reasoning underlying clinical recommendations. It is anticipated — though not yet empirically confirmed in dentistry — that such documentation would strengthen the quality of shared decision-making in dental practice.

### 4.3 Improving treatment adherence and self-management

Drawing on evidence from medical OpenNotes programs, in which survey data suggest self-reported improvements in medication adherence and quasi-experimental data indicate an association between note sharing and improved adherence (8, 9), analogous benefits may be anticipated in dentistry — particularly for self-management-dependent conditions such as periodontitis and orthodontic treatment — though this extrapolation remains to be validated in dental practice settings.

### 4.4 Enhancing access and continuity of care

Delayed access to dental care is a recognized public health challenge, particularly in publicly funded health systems where referral backlogs and waiting times for specialist or surgical care are common. Structured referral communication and triaging have been shown to reduce waiting times for elective surgical procedures (10). OpenNotes could complement these efforts by providing referring practitioners and patients with transparent, accessible documentation of clinical findings and referral rationale, facilitating more efficient triage and reducing unnecessary specialist encounters. For patients managing care across multiple providers, note access also supports continuity and reduces the risk of information loss at care transitions.

### 4.5 Supporting caregivers and vulnerable populations

Medical OpenNotes research has demonstrated particular benefits among family caregivers, who reported that note access enhanced their ability to support patients in managing complex treatment regimens (3). In dentistry, this benefit may be especially relevant for caregivers of pediatric patients, elderly individuals with cognitive impairment, and patients with physical or intellectual disabilities. For these populations, caregiver access to dental records could meaningfully enhance care coordination and advocacy. Three hypothetical clinical scenarios—periodontal self-management support, caregiver-facilitated orthodontic follow-up, and teledentistry-supported non-surgical caries management—illustrating how these anticipated benefits might be realized in practice are presented in Box 1.

Box 1 **Illustrative clinical scenarios for OpenNotes in dental practice.**

**Table:** 

The following scenarios are hypothetical illustrations of how OpenNotes might be applied in common dental contexts. They are intended to assist readers in visualising practical implementation and are not based on empirical data from dental OpenNotes programmes.
Scenario 1: Periodontal Disease Management and Self-Management Support
A 52-year-old patient with Stage III Grade B generalised periodontitis is enrolled in a structured periodontal treatment programme at an academic dental clinic with OpenNotes capability. Following each appointment, the patient receives electronic access to the clinician's visit note, which includes: probing depth and bleeding-on-probing data from the full-mouth periodontal chart; a brief lay-language summary of findings (e.g., “gum bleeding has reduced from 38% to 19% of sites since your last visit”); specific self-care instructions; and the treatment plan for the next appointment. Between visits, the patient reviews the notes using a patient portal, compares current probing data with previous charts, and uses this information to focus brushing and interdental cleaning in sites previously flagged as problematic. At the next visit, the patient reports several observations consistent with improved plaque control at specific teeth and asks a specific question about a notation in the previous note.
Anticipated benefits: By analogy with medical evidence on adherence improvement (2, 3), note access in this context may reinforce self-care motivation, improve recall of clinician instructions, and support a more collaborative, data-informed therapeutic relationship. The patient's active engagement with their own periodontal data may also support earlier identification of recurrent disease between scheduled monitoring appointments.
Scenario 2: Orthodontic Treatment Follow-Up and Caregiver Engagement
A 14-year-old patient is undergoing fixed orthodontic treatment. The patient's parent, who manages appointment scheduling and home-care supervision, is designated as an authorised caregiver with access to the patient's notes under the clinic's proxy consent framework. Following each adjustment appointment, the clinician's note includes: a structured assessment of compliance with wear of removable appliances (if relevant); a record of the specific tooth movements planned; any identified risks (e.g., demineralisation risk at specific sites); and written instructions for the interval between appointments. The parent reviews the note at home, identifies a notation about increased demineralisation risk at the upper front teeth, and reinforces the use of fluoride toothpaste and reduces acidic drink consumption as instructed. At the next appointment, the clinician notes that the patient's oral hygiene has measurably improved in the previously affected area.
Anticipated benefits: Caregiver-facilitated note access may improve domestic implementation of clinical instructions and reduce the rate of treatment complications attributable to non-compliance. The availability of written records reviewed at a time of the family's choosing — rather than exclusively during the appointment — may be particularly valuable for adolescent patients where parental involvement in clinical communication is constructive but time-constrained.
Scenario 3: Non-Surgical Caries Management Combined with Teledentistry
A 7-year-old child in a rural community attends a paediatric dental visit at a health centre with limited specialist access. The treating dentist identifies two early-stage primary molar caries lesions and — in consultation with the parent — elects a non-surgical management approach using silver diamine fluoride (SDF), combined with a remineralisation protocol and dietary counselling. The dentist documents the clinical findings, the rationale for non-surgical management, the expected outcomes (including tooth darkening), the home-care protocol, and the criteria that would indicate progression to restorative intervention. The parent receives access to the clinical note via a patient portal. Using an integrated teledentistry feature, the parent photographs the affected teeth monthly and submits images for remote review. The dentist adds brief monitoring notes to the patient record after each review, accessible to the parent in real time.
Anticipated benefits: The integration of OpenNotes with teledentistry monitoring in this scenario constitutes a comprehensive patient engagement platform for non-surgical caries management. Transparent documentation of the management rationale may reduce parental anxiety about non-restorative treatment; access to monitoring notes may improve engagement with the home-care protocol; and the combination of remote monitoring with record access may support earlier escalation to restorative care when indicated by objective image review. This scenario illustrates the synergistic potential of OpenNotes and teledentistry identified in Section 5.3.

## 5 Enabling technologies for OpenNotes implementation in dentistry

### 5.1 Artificial intelligence and natural language processing

A principal technical barrier to dental OpenNotes is the unstructured, free-text nature of much dental documentation, which limits both interoperability and the capacity for automated patient-friendly transformation. Advances in artificial intelligence (AI) and natural language processing (NLP) offer promising pathways to address this limitation. Chuang et al. (11) demonstrated that large language models, specifically Generative Pretrained Transformer 4, could be used to generate synthetic clinical notes for fine-tuning a RoBERTa model trained to extract structured diagnostic information from dental records, achieving high accuracy for periodontal diagnosis extraction across dimensions of stage, grade, and extent. Comparable approaches have demonstrated the feasibility of machine learning-based information extraction from unstructured inpatient medical records (12).

These findings suggest that AI-driven NLP tools could automate the conversion of unstructured dental notes into structured, interoperable data formats suitable for patient-facing presentation. Additionally, large language models may facilitate the automated generation of patient-friendly summaries—translating technical dental documentation into accessible language without requiring clinician reformulation of each note. This capability could substantially reduce the additional clinician workload that represents a primary concern among practitioners considering OpenNotes adoption.

Several important limitations of AI and NLP tools in patient-facing applications must be acknowledged. First, the accuracy of automated note summarization and language simplification tools has not yet been validated for dental clinical use; errors in patient-facing summaries could result in misunderstanding, unnecessary anxiety, or inappropriate health behaviors. Second, machine learning models trained on existing dental records may reflect and perpetuate existing biases related to patient demographics, practice setting, or documentation conventions, potentially producing inequitable outputs. Third, the legal liability implications of AI-generated patient-facing content remain unresolved in most jurisdictions: it is unclear whether responsibility for an inaccurate automated summary would fall on the treating clinician, the institution, or the software developer. These considerations do not preclude the development of AI-assisted OpenNotes tools in dentistry, but they underscore the necessity of rigorous clinical validation, transparent audit trails, and clear governance frameworks before deployment in patient-facing contexts.

### 5.2 Dental data infrastructure: BigMouth and interoperability

The development of the BigMouth dental data repository, which aggregates electronic health record data from over 4.5 million patients across 11 U.S. academic institutions, represents a significant step toward the standardized, interoperable dental data infrastructure that OpenNotes implementation requires (13). BigMouth has established a data governance framework facilitating cross-institutional data sharing and collaborative research. As this infrastructure matures, it may provide a technical foundation for scalable patient-facing note access across academic dental networks, beginning with the pilot implementation approach proposed in Section 6.

### 5.3 Teledentistry and remote patient engagement

Teledentistry—encompassing asynchronous image-based consultation, synchronous video encounters, and remote monitoring of oral health status—is highly complementary to OpenNotes as a patient engagement strategy. Both approaches leverage digital communication infrastructure to extend the clinical encounter beyond the physical confines of the dental operatory, supporting patient access and engagement between visits (14). In the context of minimally invasive management of early carious lesions, for example, teledentistry-enabled remote monitoring combined with OpenNotes-facilitated patient access to monitoring records could constitute a comprehensive patient engagement platform for non-surgical caries management, including home-based application of agents such as silver diamine fluoride (15). The integration of teledentistry with transparent note-sharing thus represents a potentially transformative model for patient-centered dental care.

## 6 Implementation framework

The successful integration of OpenNotes into dental practice as a patient engagement strategy requires a phased, multi-level implementation approach that addresses the technical, educational, operational, and legal dimensions identified in preceding sections (Figure 1).

**Figure 1:**
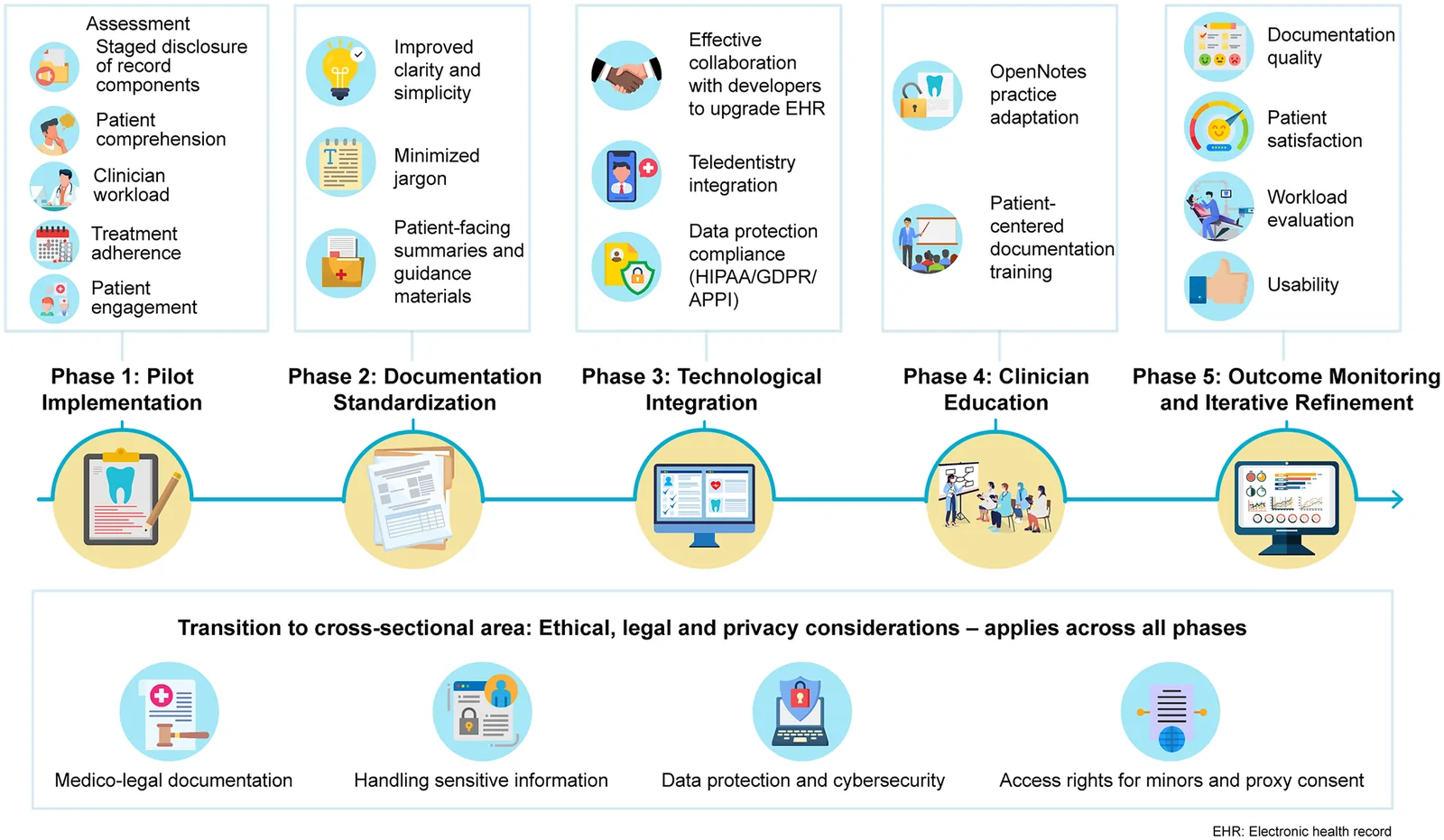
Proposed phased implementation framework for OpenNotes in dentistry. The framework comprises five sequential phases connected by right-pointing directional arrows indicating temporal progression and interdependency. Phase 1 (Pilot Implementation): staged-disclosure pilot programs in academic dental institutions; key indicators include patient engagement, patient comprehension, treatment adherence, and clinician workload. Phase 2 (Documentation Standardization): patient-centered documentation templates emphasizing improved clarity and simplicity, minimized jargon, and patient-facing summaries and guidance materials. Phase 3 (Technology Integration): collaboration with software developers to upgrade dental EHR platforms, integration with teledentistry, and compliance with data protection regulations (HIPAA, GDPR, APPI). Phase 4 (Clinician Education): OpenNotes practice adaptation and training in patient-centered documentation. Phase 5 (Outcome Monitoring and Iterative Refinement): continuous monitoring of documentation quality, patient satisfaction, clinician workload, and system usability. A dashed feedback arrow from Phase 5 to Phase 1 indicates iterative refinement of implementation approaches based on monitored outcomes. Ethical, legal, and privacy safeguards (Section 6.4)—encompassing medico-legal documentation, handling of sensitive information, data protection and cybersecurity, and access rights for minors and proxy consent—are represented as a horizontal band spanning all phases. EHR, electronic health record.

### 6.1 Phase 1

Pilot Implementation: Initiation within academic dental institutions or hospital-based dental departments provides controlled environments with existing EHR infrastructure, regulatory compliance frameworks, and research capacity. Pilots should prospectively collect outcome data including patient-reported engagement, comprehension, treatment adherence, clinician workload, and documentation quality (3). Specific attention should be directed to patient populations with complex treatment needs, including periodontal disease, implant therapy, and multi-disciplinary cases, where engagement benefits may be most pronounced. International experience with patient record access systems, including the Swedish Journalen model, supports the potential value of a staged-disclosure approach to dental OpenNotes implementation. Under this model, initial access would be limited to record components that are lower in clinical sensitivity and more readily interpretable by patients without professional mediation—such as oral hygiene instruction records, appointment summaries, and patient-reported outcome measures—before progressively extending access to more technically complex or image-dependent documentation, including periodontal charts, radiographic reports, and specialist referral letters. This approach allows patients, clinicians, and institutions to develop familiarity with record-sharing processes before encountering documentation more likely to generate anxiety or require contextualization, and provides a mechanism for identifying implementation challenges at lower-risk stages before broader rollout. Pilot programs should prospectively evaluate whether staged access produces differential engagement or comprehension outcomes compared with unrestricted access.

### 6.2 Phase 2

Documentation Standardization: Parallel to pilot programs, the development of patient-centered documentation templates for common dental encounter types—new patient examinations, periodontal assessments, treatment planning consultations, and recall appointments—would facilitate both note readability and AI-assisted processing. Templates should minimize unexplained technical abbreviations, incorporate embedded glossaries or lay-language alternatives, and include standardized patient-facing summaries of treatment options discussed and decisions made.

### 6.3 Phase 3

Technology Integration: Collaboration among dental software developers, academic institutions, and regulatory bodies is necessary to develop and certify patient-facing note access functionality within dental EHR platforms. Integration with teledentistry platforms and patient engagement portals should be prioritized to create seamless digital engagement ecosystems. Compliance with data protection regulations—including HIPAA, GDPR, and Japan's Act on the Protection of Personal Information (APPI) and Guidelines on Security Management of Medical Information Systems—must be ensured through robust patient authentication, consent management, and access control mechanisms.

### 6.4 Ethical, legal, and privacy considerations

The implementation of OpenNotes in dentistry raises distinct ethical and legal considerations that do not have direct parallels in medical settings and must be explicitly addressed in any implementation framework. Medico-legal implications of elective care documentation:

Unlike many medical interventions, dental procedures are predominantly elective, and the clinical record serves as a primary evidentiary document in the event of patient complaints, insurance disputes, or litigation. Patients' unrestricted access to notes documenting treatment alternatives, clinician recommendations, and consent processes may alter the dynamics of disputes arising from elective decisions. Dental practitioners and institutions should ensure that documentation practices are legally robust before and after implementing patient access, and should seek advice from professional indemnity organizations regarding the implications of transparent note-sharing in their jurisdiction. Insurance and third-party documentation:

Dental records frequently serve dual functions: as clinical care records and as insurance claim documentation. In jurisdictions where dental benefit schemes audit clinical records, patient access may raise questions about the appropriate handling of records that contain financially sensitive information, or where the requirements of insurance documentation differ from those of patient-centered narrative. Institutions implementing OpenNotes should develop clear policies distinguishing between records maintained for clinical purposes and those prepared for reimbursement. Data protection and cybersecurity:

Patient-facing note access requires robust digital infrastructure and information governance. Relevant regulatory frameworks include the U.S. Health Insurance Portability and Accountability Act (HIPAA), the European General Data Protection Regulation (GDPR), and Japan's Act on the Protection of Personal Information (APPI) together with the Ministry of Health, Labour and Welfare's Guidelines on the Security Management of Medical Information Systems. Implementation must incorporate strong patient authentication, end-to-end data encryption, access logging, breach notification procedures, and regular security auditing. Dental practices, which often operate as small independent units with limited IT infrastructure, may be particularly vulnerable to cybersecurity risks and should receive targeted support. Access rights for minors and proxy consent:

Dental care frequently involves pediatric patients and individuals for whom proxy or caregiver consent is required. Clear protocols are needed governing the respective access rights of patients, parents or guardians, and appointed caregivers, and the circumstances under which access may be restricted in the interests of patient welfare.

Documentation candor: Evidence from medical settings suggests that patient access may in some contexts alter clinician documentation behavior, with practitioners reporting avoidance of speculative or sensitive entries (18). In dentistry, where records routinely document early-stage or uncertain findings — such as incipient carious lesions under surveillance, or provisional diagnoses pending specialist referral — the implications for documentation completeness and clinical accuracy warrant careful monitoring in pilot programs.

### 6.5 Phase 4

Clinician Education: Both undergraduate dental curricula and continuing professional development programs should incorporate training in patient-centered documentation, transparent clinical communication, and the ethical dimensions of note-sharing. Clinicians must be prepared not only with technical skills but with a conceptual reorientation toward documentation as a communicative and therapeutic tool rather than solely an administrative and medicolegal record.

### 6.6 Phase 5

Outcome Monitoring and Iterative Refinement: Continuous monitoring of patient-reported outcomes, clinician experience, documentation quality, and system usability should inform iterative refinement of implementation approaches. Structured feedback mechanisms for both patients and providers, combined with regular audit processes, will be essential to sustain and improve OpenNotes programs over time.

## 7 Discussion

Evidence from medical OpenNotes programs demonstrates improvements in treatment adherence, shared decision-making, and patient empowerment. Dental care, characterized by complex treatment alternatives and elective decision points, may represent an especially suitable domain for OpenNotes-driven engagement. Key barriers include procedure-centric documentation, specialized terminology, fragmented electronic health record adoption, and privacy regulations. Advances in natural language processing, machine learning, and teledentistry offer potential pathways to overcome these challenges.

Several important limitations of the present analysis merit explicit acknowledgment. First, no primary data from dental OpenNotes programs currently exist; the evidence base for anticipated benefits in dentistry is derived by analogy from medical contexts, and extrapolation across these domains carries inherent uncertainty. Patient populations, documentation cultures, and care delivery models differ substantially between medicine and dentistry, and the magnitude of engagement benefits in dental OpenNotes programs may differ from those observed in medical settings.

Second, the present review employed a narrative rather than systematic methodology. A comprehensive systematic search of the literature was not conducted, and the evidence synthesis presented herein may be subject to selection bias. Future work should employ systematic or scoping review methodology, with transparent search strategies and inclusion criteria, to provide a more rigorous evidentiary foundation.

Third, the challenges associated with dental EHR fragmentation, particularly outside the U.S. academic context, may be more pronounced than the present analysis suggests. The feasibility of the proposed implementation framework will vary substantially across healthcare systems, practice settings, and regulatory environments.

Furthermore, the present review has focused primarily on the U.S.-derived OpenNotes literature; the broader international evidence base for Online Record Access (ORA), including studies from Nordic healthcare systems where patient access to electronic records is already well-established, has not been systematically incorporated and represents an important avenue for future review.

Fourth, while the potential for AI and NLP tools to support dental OpenNotes implementation is theoretically compelling, the clinical validation of such tools for patient-facing applications remains at an early stage. Concerns regarding accuracy, bias, and the appropriate scope of automated clinical language transformation warrant careful investigation before deployment in patient-facing contexts.

Patient engagement is fundamental to the quality and ethics of dental care. OpenNotes represents a structurally simple but potentially transformative mechanism for operationalizing engagement through documentation transparency. The elective, decision-intensive nature of dental care creates particular opportunities for engagement benefits analogous to and potentially exceeding those demonstrated in medical OpenNotes programs. Barriers specific to dental documentation—including technical language, image-dependent records, and EHR fragmentation—are real but addressable through advances in AI-assisted documentation, standardization efforts, and teledentistry integration.

Clinician acceptance of OpenNotes is a critical determinant of OpenNotes implementation success and must be addressed directly. Evidence from medical settings demonstrates that clinicians consistently overestimate the burden of OpenNotes before implementation: in the foundational OpenNotes pilot, fewer than one in twenty physicians reported longer consultations, fewer than one in ten reported increased after-hours queries, and no physician elected to discontinue at the study's conclusion (2). Nevertheless, some concerns — particularly regarding documentation candor and the risk of altered note-writing behavior — have been substantiated in qualitative research from primary care settings (18). In dentistry, the following specific concerns merit attention: (1) the increased time required to explain or contextualize technical findings in response to patient queries; (2) the risk that clinicians modify note content — for example, avoiding documentation of uncertain radiographic findings — to reduce anticipated patient anxiety; and (3) the potential for patient-accessible records to generate medico-legal vulnerability in elective care contexts. These concerns should be taken seriously in the design of dental OpenNotes programs, addressed through targeted clinician education, supported by clear institutional guidance, and evaluated empirically through prospective workload measurement in pilot studies. The potential of AI-assisted note summarization to generate patient-facing lay-language summaries from existing clinical notes — without requiring clinicians to reformulate documentation — represents a practical strategy for reducing the workload dimension of this concern (see Section 5.1).

Several priority areas for future research can be identified from this review. First, controlled pilot implementation studies in academic dental settings are urgently needed. Such studies should prospectively measure patient-reported engagement and comprehension, treatment adherence (particularly for self-management-dependent conditions including periodontitis and orthodontic treatment), clinician workload and documentation behavior, and patient-reported outcomes including satisfaction and health literacy. Periodontal disease management, complex restorative treatment planning, and caregiver-facilitated pediatric care are the highest-priority contexts for initial pilot work, given the anticipated magnitude of engagement benefits in these settings. Second, AI-assisted note simplification tools specifically validated for dental documentation require development and rigorous evaluation. Studies should assess the accuracy of AI-generated lay-language summaries against the source clinical records, examine whether simplified summaries alter patient behavior in intended and unintended ways, and investigate how the responsibility for errors in automated summaries should be allocated across clinician, institution, and software developer. Third, longitudinal outcome studies are needed to determine whether the engagement improvements anticipated from dental OpenNotes translate into measurable differences in clinical outcomes—including periodontal disease progression, treatment completion rates, and recall attendance—over clinically meaningful time periods. Fourth, comparative international research should examine how differences in legal frameworks, patient expectations, and health system organization affect the feasibility and impact of dental OpenNotes implementation across different settings, including Japan, where the regulatory environment (APPI; Ministry of Health, Labour and Welfare guidelines) and dental insurance system present distinct contextual considerations. Fifth, qualitative research with both dental clinicians and patients—including those from traditionally underserved populations—is needed to understand the attitudes, information needs, and implementation concerns that will shape adoption in real-world dental settings.

The proposed phased implementation framework, beginning with controlled pilots in academic dental settings, offers a pragmatic pathway toward generating the dental-specific evidence base that the field currently lacks. Realizing the patient engagement potential of dental OpenNotes will require coordinated investment in technology infrastructure, clinical education, and regulatory compliance—investments that are justified by the prospect of more informed, empowered, and engaged dental patients.
